# Inferior olive CRF plays a role in motor performance under challenging conditions

**DOI:** 10.1038/s41398-018-0145-3

**Published:** 2018-05-25

**Authors:** Gili Ezra-Nevo, Naama Volk, Assaf Ramot, Claudia Kuehne, Michael Tsoory, Jan Deussing, Alon Chen

**Affiliations:** 1 Department of Neurobiology, Weizmann Institute of Science, Rehovot, 76100 Israel; 2 Department of Stress Neurobiology and Neurogenetics, Max Planck Institute of Psychiatry, 80804 Munich, Germany; 3 Department of Veterinary Resources, Weizmann Institute of Science, 76100 Rehovot, Israel

## Abstract

A well-coordinated stress response is pivotal for an organisms’ survival. Corticotropin-releasing factor (CRF) is an essential component of the emotional and neuroendocrine stress response, however its role in cerebellar functions is poorly understood. Here, we explore the role of CRF in the inferior olive (IO) nucleus, which is a major source of input to the cerebellum. Using a CRF reporter line, in situ hybridization and immunohistochemistry, we demonstrate very high levels of the CRF neuropeptide expression throughout the IO sub-regions. By generating and characterizing IO-specific CRF knockdown and partial IO-CRF knockout, we demonstrate that reduction in IO-CRF levels is sufficient to induce motor deficiency under challenging conditions, irrespective of basal locomotion or anxiety-like behavior. Furthermore, we show that chronic social defeat stress induces a persistent decrease in IO-CRF levels, and that IO-CRF mRNA is upregulated shortly following stressful situations that demand a complex motor response. Taken together our results indicate a role for IO-CRF in challenge-induced motor responses.

## Introduction

When a situation is perceived as stressful, the brain activates many neuronal circuits, linking centers involved in sensory, motor, neuroendocrine, cognitive, and emotional functions in order to adapt to the demand. There is substantial evidence to suggest that inappropriate regulation, disproportional intensity, or chronic and/or irreversible activation of the stress response is linked to the etiology and pathophysiology of anxiety and depression^1–5^.

Corticotropin-releasing factor (CRF) and its type 1 receptor (CRFR1) have a unique role in mediating behavioral and physiological responses to diverse stressors. These systems may be particularly important in situations where an organism must mobilize not only the hypothalamic–pituitary–adrenal (HPA) system, but also the central nervous system in response to environmental challenge^1,2,5^. Interestingly, it has been repeatedly demonstrated that CRF is prominently expressed in the inferior olive (IO) of various species from rodents to primates, as well as localized in the fibers descending from the IO, namely, the climbing fibers (CFs)^6–17^.

The IO, which constitutes one of the two major inputs to the cerebellum and to Purkinje cells (PCs) in particular, is best known for its role in motor adjustment, coordination, balance, and learning of motor skills^18,19^. Interestingly, altered cerebellar connectivity and vestibular problems were found in PTSD patients and several forms of anxiety disorders, however the interaction between acute or chronic stress and motor performance is still poorly understood^20–23^.

In this study, we explored the contribution of CRF in the IO to the well-orchestrated stress response of the adult mouse. We show that site specific reduction of IO-CRF in the adult mouse was sufficient to induce a challenge-induced motor deficit, without affecting baseline motor activity. We show that chronic social defeat stress (CSDS) induces a persistent decrease in IO-CRF levels. We further present evidence that IO-CRF is regulated following specific stressors that comprise a motor challenge indicating CRF “participation” in coping with these challenges.

## Materials and methods

### Animals and experimental groups

Detailed description of the animals and experimental groups used in this study can be found in SI Methods.

### Behavioral studies

All behavioral paradigms were performed as previously described^24–26^ with modifications. For a detailed description of the behavioral tests, see SI Methods.

### Immunohistochemistry and in situ hybridization

In situ hybridization^27–29^ and immunohistochemistry^26,30^ were performed as described. For a full description of the protocols and antibodies used, see SI Methods.

### CLARITY and whole-brain imaging

The CLARITY method used here was based on protocols reported by Ye et al.^31^ and modified. A detailed description can be found in the SI Methods.

### Viral constructs and stereotaxic injections

All constructs were assembled by using standard cloning methods and confirmed by DNA sequencing and described in^27,28,32^. Stereotaxic injections were performed as described by Ramot et al.^26^. For a detailed description, see SI Methods.

### mRNA extraction and quantification with qRT-qPCR

Preparation and quantification of mRNA was performed as previously described^30^. A full description can be found in SI Methods.

### Statistical analysis

Results are expressed as mean ± SEM. Automated analysis was used whenever possible, including cell counting and behavioral measurements. Samples that were 2 SD above or below the group mean were excluded. Statistical analysis was conducted using SPSS software (SPSS Inc, Chicago, IL). Following a test of normality, statistical significance was determined by Student’s *t*-test or one-way analysis of variance (ANOVA) followed by Fisher’s LSD post hoc test, or by non-parametric test (Mann–Whitney *U* test; MW) or two-way repeated measures ANOVA, when appropriate. No differences in variance between groups were statistically significant. Statistical tests were two-sided. *p* < 0.05 was considered significant.

## Results

### CRF is expressed throughout the IO and highly expressed in both the mouse and human IO

**Fig. 1:**
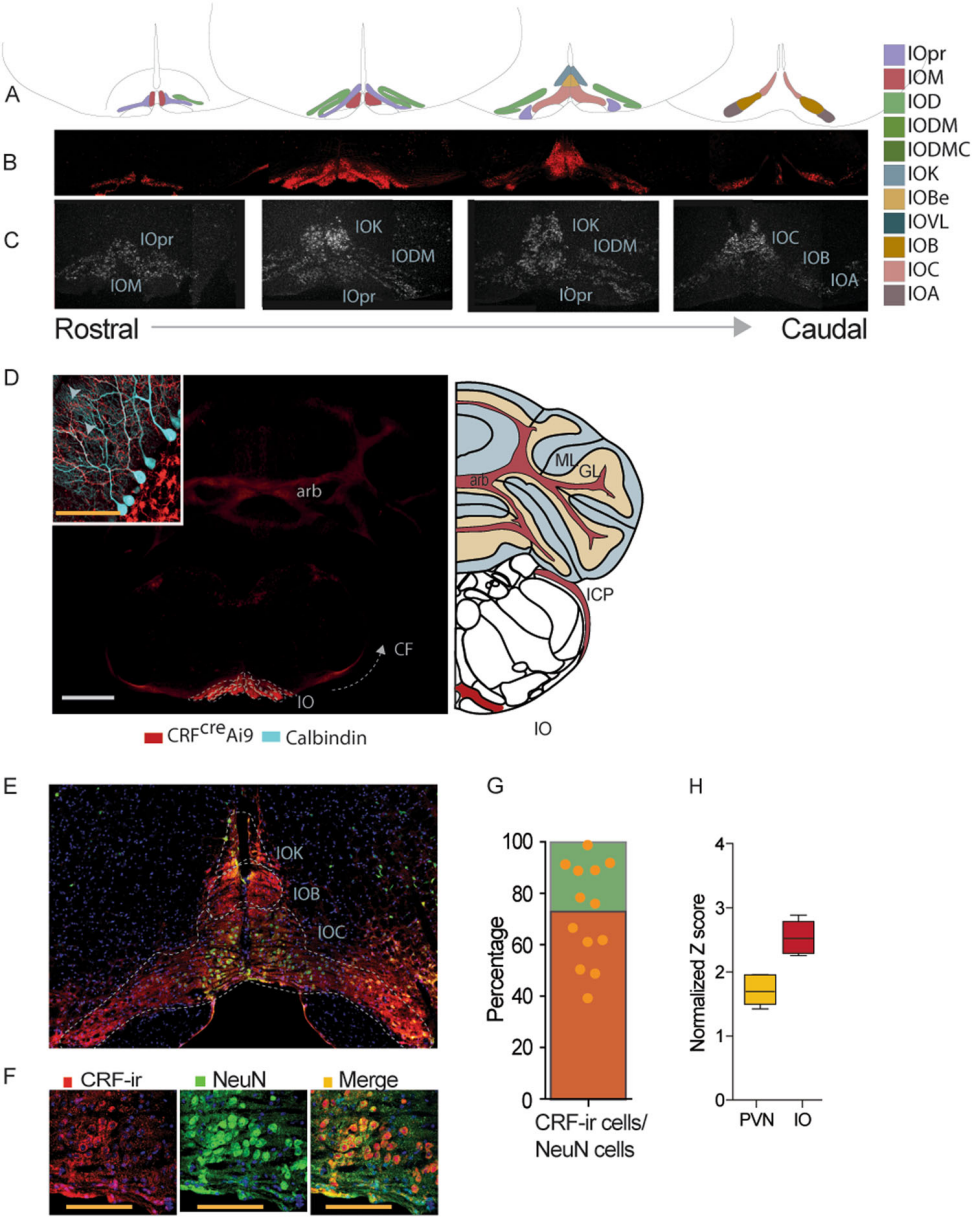
CRF is highly expressed in the IO of the mouse. **a** Schematic coronal representation of four slices of the IO and its sub-nuclei. IOpr IO principal nucleus, IOD IO dorsal nucleus, IODM IO dorsomedial cell group, IOM IO medial nucleus, IODMC IO dorsomedial cell column, IOK cap of Kooy medial nucleus, IOBe IO beta subnucleus, IOV IO ventral nucleus, IOB IO subnucleus B medial nucleus, IOC IO subnucleus C medial nucleus, IOA IO subnucleus A medial nucleus. **b** The IO of a CRF-*Cre* mouse line crossbred with conditional tdTomato mouse line (CRF-tdTomato; red). tdTomato signal indicates CRF expression, which is seen from the most rostral part to the most caudal structures of the IO. **c** CRF In situ hybridization of wild type (WT) mouse. Dark field images show high CRF mRNA signal throughout the IO of the mouse, compatible with expression patterns seen in the reporter line. **d** Coronal section of a CRF-tdTomato mouse stained for calbindin (cyan), a Purkinje cell (PC) marker. Image depicts climbing fibers (CF) expressing tdTomato (red), indicating CRF expressing cells reach the molecular layer of the cerebellar cortex (gray arrows indicate tdTomato expressing fibers in close proximity to PCs dendritic tree). ICP inferior cerebellar peduncle, ML molecular layer, GL granular layer, arb arbor vitae, IOK cap of Kooy medial nucleus, IOB IO subnucleus B medial nucleus, IOC IO subnucleus C medial nucleus Gray scale bar (bottom) = 1000 μm, orange scale bar (top left) = 100 μm. **e** Representative image of mouse IO stained with CRF antibody (red) and neuronal marker (NeuN; green), along with Hoechst staining (blue). **f** Representative Hoechst stained (blue) images of IO cell bodies co-stained for CRF (red) and NeuN (green), and a merged image. Orange scale bar = 100 μm. **g** Summary of IO NeuN-positive cells stained for CRF. 70% of NeuN stained cells (green bar), are co-stained for CRF (orange bar; based on the staining of 6 and 7 IO sections from 2 mice, a total of 13 sections, each dot in the graph represents an IO slice). **h** Relative quantity of CRF mRNA from humans obtained from the Allen brain institute for Brain Science. Data (from six donors) shows that in humans, CRF expression in the IO is even higher than in the paraventricular nucleus of the hypothalamus, the hallmark region for CRF expression

**Fig. 2:**
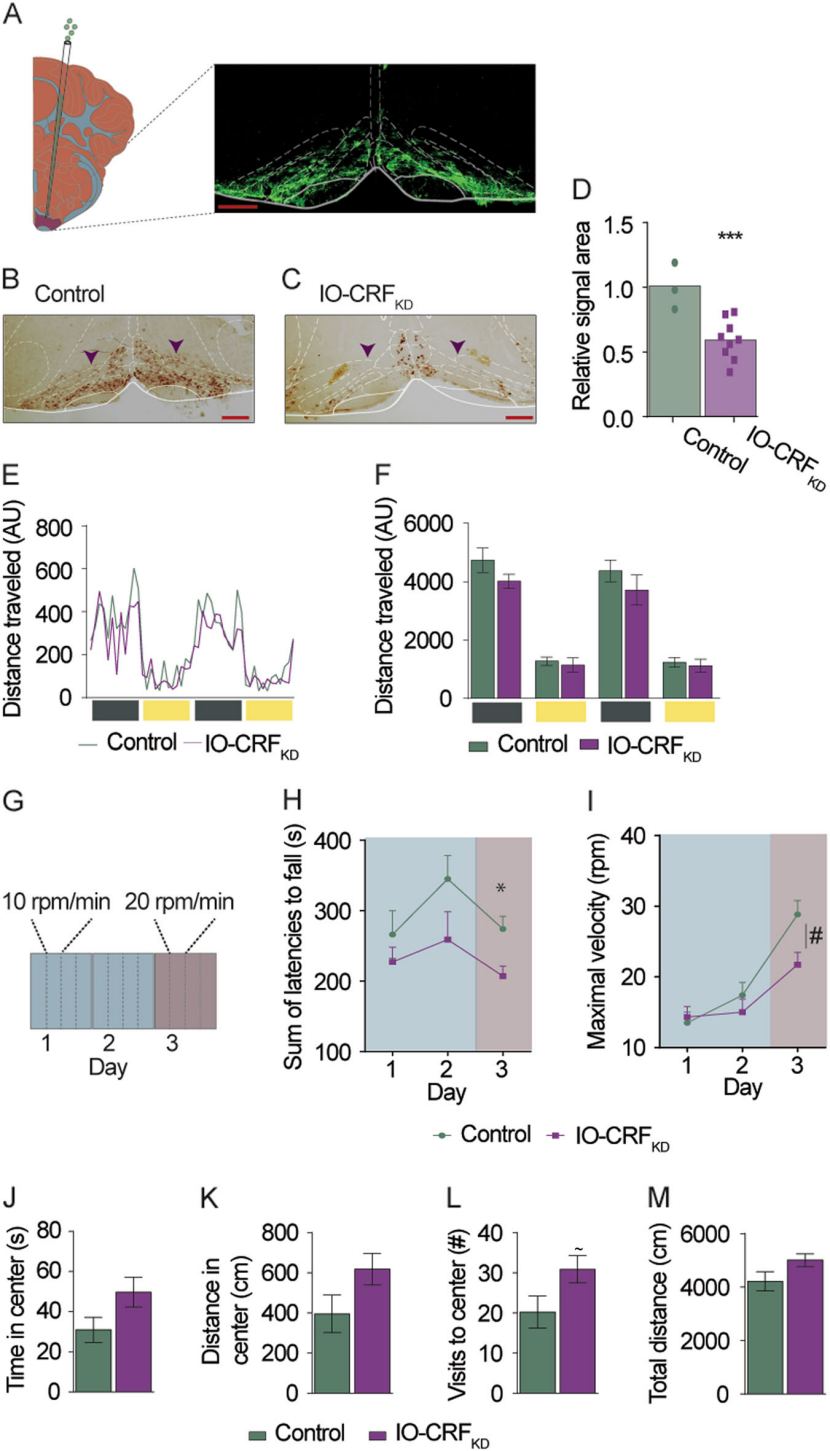
Intact home cage locomotion but impaired challenge-induced motor performance in IO-specific CRF KD mice. Knockdown (KD) of CRF specifically in the IO of adult mice is sufficient to impair motor performance on the rotarod. **a** Schematic illustration of lentiviral injection and a representative confocal microscope image of a virally infected IO of an adult C57 mouse. Red scale bar = 250 μm. In situ hybridization using probes for CRF showing the IO of mice injected with **b** control virus and **c** KD virus. Red scale bar = 250 μm. **d** Relative CRF in situ hybridization signal area of KD and control injected IO (each dot represents a slice, *n* = 3, 9 slices). **e**, **f** Baseline locomotion of IO-CRF_KD_ mice and control mice was measured using inframot. KD mice did not differ from control mice in home-cage locomotion (*n* = 7, 9). **g** Schematic illustration of the rotarod protocol used. Mice underwent 2 days of training with the rotarod accelerating from 0 to 40 rpm in 4 min (inclination of 10 rpm/min) then on the 3rd day, the rotarod was accelerated from 0 to 40 in 2 min (20 rpm/min). Each day, mice were subjected to four trials with a 2-min break in between. Sum of latencies to fall and maximal velocity reached were measured. IO-CRF_KD_ and control mice motor performance was tested using the rotarod. **h** Although on average IO-CRF_KD_ lasted less time on the rotarod compared to control mice, no overall significant differences were detected between the groups, but significant difference in latency to fall was seen at the highest velocity (20 rpm; *n* = 10, 9). **i** Maximal velocity reached on the rotarod was significantly higher for control than KD mice (*n* = 10, 9). **j**–**m** IO-CRF_KD_ and control mice were tested for anxiety-like behavior using the open field (OF) test. No differences in anxiety-like behavior were detected between KD and control mice (*n* = 9, 9). Data are presented as mean + SEM. ^~^*p* < 0.07, *p < 0.05, ***p < 0.005. Significant interaction (time × group) marked as ^#^*p* < 0.01

We validated CRF expression and sub-nuclei distribution in the IO using the CRF-*Cre* mouse line^33^ crossbred with mice conditionally expressing tdTomato^34^ (CRF-tdTomato; Fig. 1a–c, Supplementary Fig.1B), as well as in situ hybridization (ISH) for CRF mRNA (Fig. 1c, Supplementary Fig.1C). The tdTomato reporter signal produced by the CRF positive IO neurons was clearly evident throughout the IO and in the CF reaching the cerebellum, as seen in Fig. 1a, b, d, and more elaborately in Supplementary Fig.1A-C. Supplementary Movie 1 of a CRF-tdTomato mouse brain imaging depicts the profuse tdTomato positive staining of fibers going from the IO to the cerebellum. CRF ISH shows a similar expression pattern (Fig. 1c). CFs originating in CRF expressing cells (showing tdTomato signal) reach the cerebellar cortex and climb over PCs (stained in Cyan; gray arrows indicate CFs in close proximity to PCs dendritic tree; Fig. 2d). We used colchicine injected mice to quantify the percentage of CRF positive neurons within the IO. Coronal brain sections from these mice were immunostained for both NeuN (green) and CRF (red; Fig. 1e, f) along with Hoechst staining (blue). This triple staining revealed that more than 70% of IO neurons express CRF (Fig. 1g). An additional assessment of the percentage of CRF-positive cells in the IO was performed using CRF-Ai9 mice with calbindin (an IO marker) IO immunostaining. This analysis yielded similar results, again indicating that ~70% of IO neurons express CRF (Supplementary Fig. 1). Lastly, data of relative expression of CRF in humans, obtained from the Allen human brain atlas, shows CRF has an extremely high expression level in the human IO (Fig. 1h; Allen Brain institute, Human Microarray data, 2016,^35^; probe 1057965). These results indicate that IO-CRF is likely to play a conserved role in both the mouse and human cerebellar system.

### Intact home cage locomotion but impaired challenge-induced motor performance in IO-specific CRF knockdown mice

We next examined whether reduced IO-CRF was sufficient to induce motor impairment. To that end, we injected lentivirus expressing previously validated shCRF (knockdown, KD) or control construct into the IO of adult C57/BL mice^27,28^; IO-CRF_KD_; Fig. 2a). Quantification of the CRF mRNA signal using ISH (Fig. 2b, c) on brain slices showed a CRF mRNA signal reduction of ~50% in the IO area of KD mice compared to controls (*t*-test, *t*_(10)_ = 3.818, *p* = 0.003; Fig. 2d).

Mice injected with the KD virus into the IO (IO-CRF_KD_) did not differ in home-cage locomotion from control-injected mice during the light or the dark phases (Fig. 2e, f). Mice were then tested on the rotarod for three consecutive days. The rotating rod was accelerated by additional 10 rpm each min (0–40 rpm in 4 min), 4 trials/day, for 2 days, followed by a third day on which the speed was accelerated by 20 rpm per min (Fig. 2c; see Materials and methods). IO-CRF_KD_ mice lasted shorter periods of time on the rotating rod, reaching significance only on day 3 (Fig. 2g). IO-CRF_KD_ reached lower maximal velocities in the course of training (two-way repeated measures ANOVA, interaction of group and training day, *F*_(2,34)_ = 6.303, *p* = 0.005, *n* = 10,9; Fig. 2h), Student’s *t*-test reveals the difference between the groups becomes significant at the 20 rpm speed (*t*_(17)_ = 2.698, *p* = 0.015, Bonferroni-corrected critical *p* = 0.0166; Fig. 2e). In order to further assess baseline gait and coordination, we tested mice on the CatWalk (Noldus). Data were collected on the following measures: base of support (BOS), print position (to evaluate gait or balance disturbance), and Regularity Index (overall coordination assessment) (Supplementary Fig. 2A-C). KD mice did not differ from control counterparts, implying that reduced IO-CRF levels are particularly relevant for motor tasks that require effort rather than for baseline locomotion (Supplementary Fig. 2B-C). When tested for anxiety-like behavior in the open-field (OF) test, IO-CRF_KD_ mice did not differ from their control littermates, except for a tendency for significance in the number of visits to the center (*p* = 0.058, Bonferroni correction critical *p* = 0.0125; Fig. 2j–m). These results suggest that reduced IO-CRF can induce a specific challenge-induced motor phenotype. Next, we employed the *Cre*-lox KO system to induce a more pronounced IO-CRF reduction.

### IO-specific partial CRF-KO is sufficient to induce motor deficit in mice

**Fig. 3:**
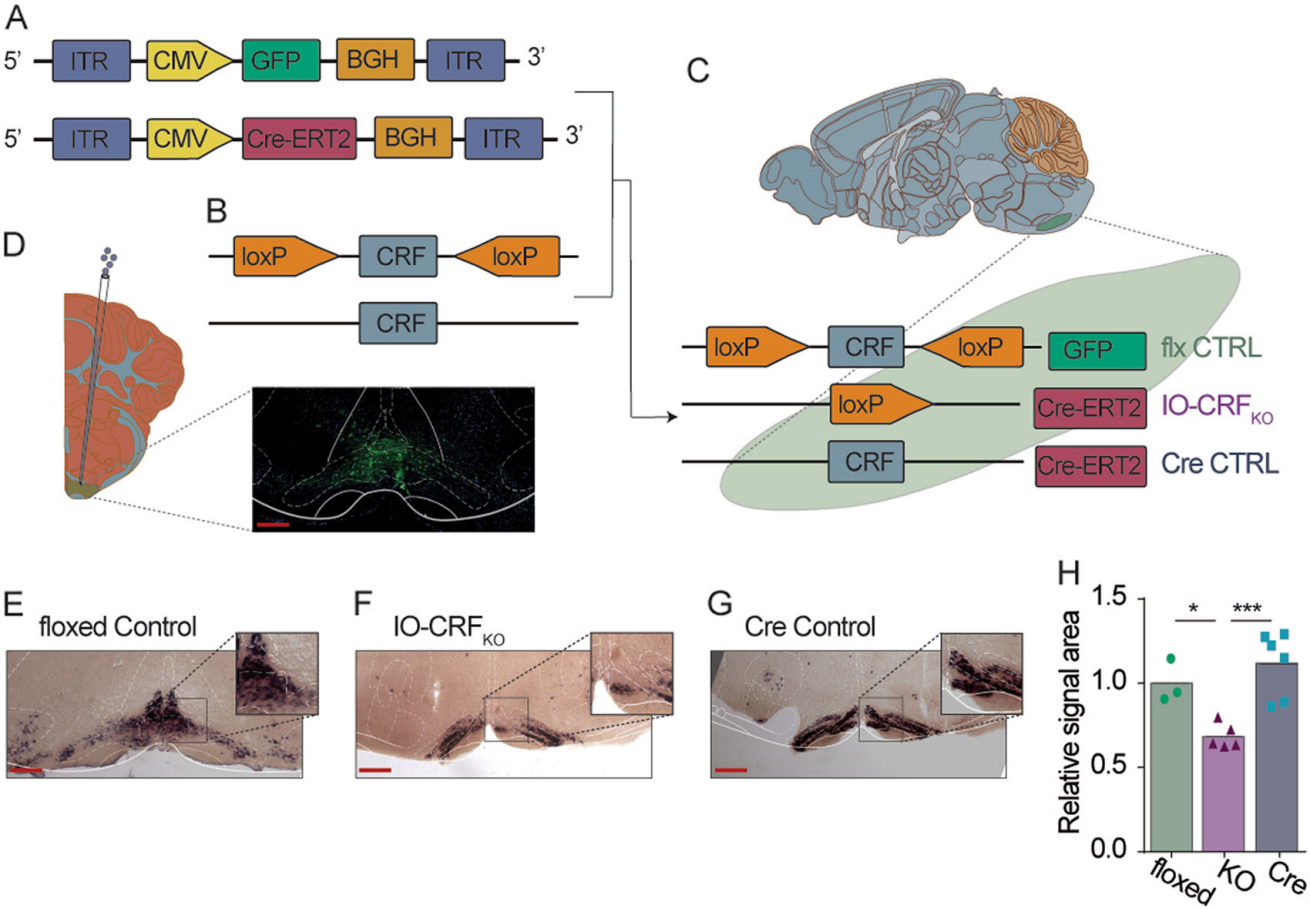
Generation of IO-specific CRF partial KO mice. Floxed CRF or wild-type mice were injected with *Cre* expressing virus to induce a site-specific partial knockout (KO). **a** Schematic illustration of tamoxifen-induced *Cre* (*Cre*-ERT2) expressing adeno-associated virus and control GFP expressing virus. **b**, **c** Viral constructs were injected to floxed CRF mice and WT mice, to obtain control for floxed CRF background, site-specific partial CRF_KO_ and control for the *Cre* activity. **d** Representative microscope image of virally infected IO of an adult mouse. Red scale bar = 250 μm. **e**–**g** In situ hybridization using probes for CRF showing **e** the IO of floxed CRF mice injected with control virus (floxed control) and **f** with *Cre*-ERT2 virus (IO-CRF_KO_) and **g** WT littermates injected with *Cre*-ERT2 virus (*Cre* control). Red scale bar = 250 μm. **h** Relative signal area of CRF in situ hybridization obtained from floxed-control mice, partial IO-CRF_KO_ and *Cre*-control (each dot represents an IO slice; *n* = 3, 5, 6 slices for floxed control, KO and *Cre* control, respectively). Post hoc Fisher’s LSD shows differences between IO-CRF_KO_ group and the two control groups, but not between control groups. *p < 0.05, ***p < 0.005

**Fig. 4:**
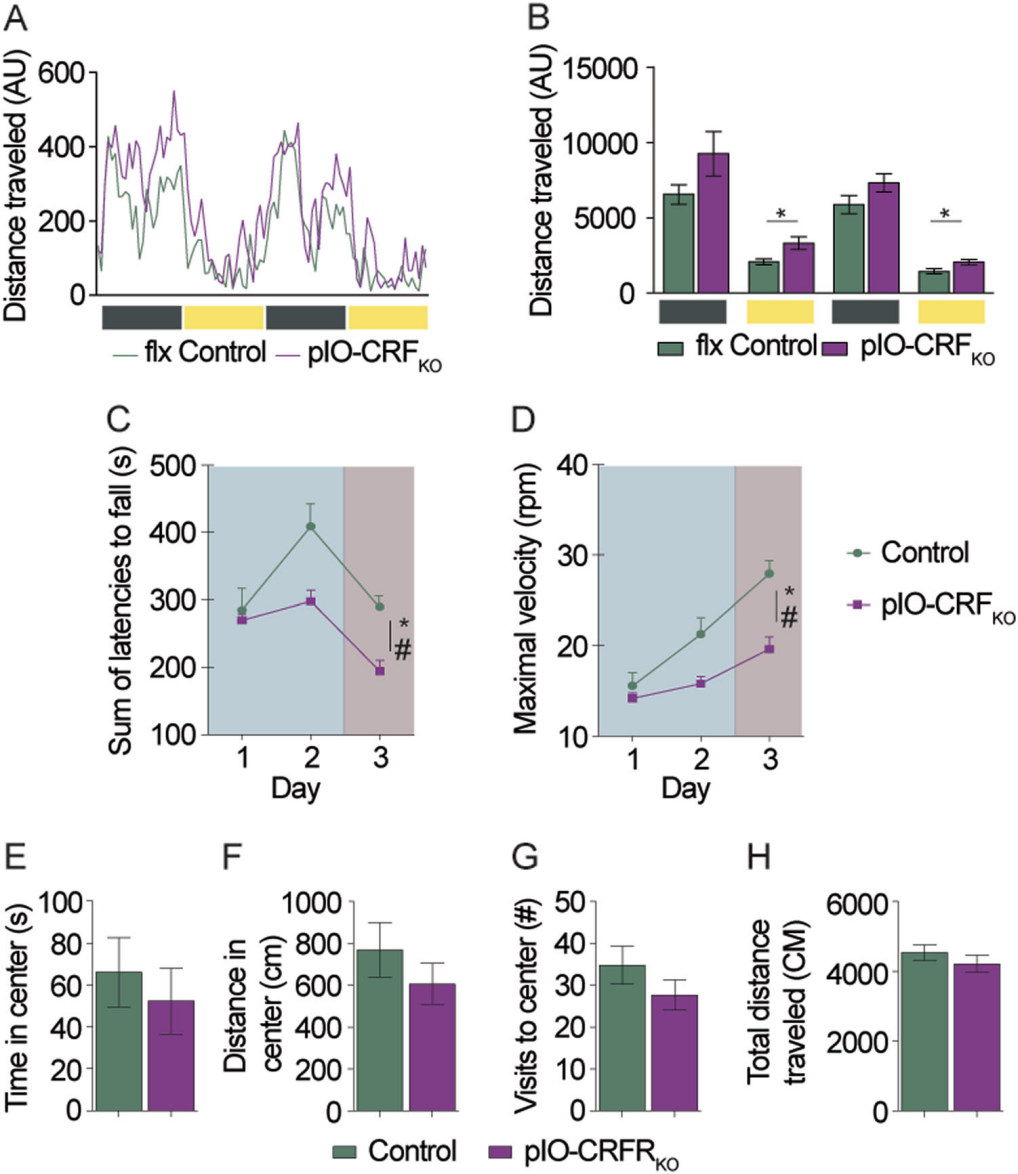
IO-specific partial CRF-KO is sufficient to induce a motor deficit in mice. **a**, **b** Baseline motor activity of control and partial IO-CRF_KO_ (pIO-CRF_KO_) mice. Groups did not differ in home cage locomotion during the dark phase, however pIO-CRF_KO_ mice had higher levels of locomotion during the light phase (*n* = 10, 8). **c**, **d** pIO-CRF_KO_ and control mice motor performance was tested using the rotarod. **c** IO-CRF_KD_ were lower than control mice in sum of latencies to fall of the rotarod (*n* = 10, 7). **d** pIO-CRF_KO_ also reached lower maximal velocities on the rotarod compared to controls (*n* = 10, 7). **e**–**h** pIO-CRF_KO_ and control mice were tested for anxiety-like behavior using the open field test. No differences in anxiety-like behavior were detected between KO and control mice (*n* = 10, 8). *p < 0.05, ***p < 0.005. Significant interaction (time × group) marked as ^#^*p* < 0.05

Tamoxifen inducible *Cre* expressing adeno-associated virus (*Cre*- ERT2) or control virus was injected into adult floxed CRF mice (see Materials and Methods and Supplementary Methods; Fig. 4a, b). In order to insure *Cre* did not affect the cells’ viability nor levels of CRF, WT littermates were also injected with *Cre*-ERT2 (Fig. 3a–c). All mice received tamoxifen solution by gavage (Fig. 3c). Location of the injection was validated using either GFP staining or staining against *Cre* (Fig. 3d). Post *Cre* induction, CRF levels were assessed using ISH on slices obtained from floxed mice injected with GFP expressing virus (floxed control; Fig. 4e), floxed mice injected with *Cre*-ERT2 (i.e., IO-CRF_KO_; Fig. 3f) and WT mice injected with *Cre*-ERT2 (*Cre* control; Fig. 3g). Floxed CRF mice injected with *Cre*-ERT2 showed significantly less CRF ISH signal compared to both control groups (one-way ANOVA, *F*_(2,11)_ = 11.931, *p* = 0.002; Post hoc Fisher’s LSD shows differences between IO-CRF_KO_ group vs. *Cre* control group, *p* = 0.001, and vs. floxed control group, *p* = 0.014; Fig. 3h). Importantly, the signal in slices obtained from *Cre*-ERT2 WT mice did not differ from that in floxed controls (Fig. 3h). Although CRF in the infected cells was completely abolished in IO-CRF_KO_ mice, we were only able to cover a moderate part of the IO using intracerebral injections, and thus we obtained only a partial IO-CRF_KO_ (pIO-CRF_KO_; ~40% reduction; Fig. 3h).

Analysis of home-cage basal activity of pIO-CRF_KO_ mice and control mice revealed a small increase in locomotor activity of pIO-CRF_KO_ mice compared to controls, only during the light phase (*t*_(16)_ = 3.120, *p* = 0.007, *t*_(16)_ = 3.05, *p* = 0.008; *n* = 10, 8, Bonferroni-corrected critical *p* = 0.0125; Fig. 4a, b). In marked contrast, when the mice were tested on a challenging motor task, the rotarod, pIO-CRF_KO_ mice performed significantly worse than control mice (Fig. 4c, d). KO mice lasted shorter periods of time and showed a more gradual improvement during training (two-way repeated measures ANOVA, main effect for group, *F*_(1,15)_ = 6.235, *p* = 0.025; interaction *F*_(2,30)_ = 3.715, *p* = 0.036, *n* = 10, 7; Fig. 4c) and reached lower maximal velocities (repeated measures ANOVA, main effect for group, *F*_(1,15)_ = 8.599, *p* = 0.01; interaction *F*_(2,30)_ = 6.169, *p* = 0.006, *n* = 10,7; Fig. 4d). To further evaluate the gait and coordination of pIO-CRF_KO_ mice, they were tested on the CatWalk (Noldus; Supplementary Fig. 2A and 3). Mice with pIO-CRF_KO_ did not differ from controls in BOS, and in spite a difference in average print position, none of the differences yielded significance following multiple comparison *p* value correction (Supplementary Fig. 3A). The Regulatory Index of KO mice did not differ from that of controls (Supplementary Fig. 3B). The pIO-CRF_KO_ group did not differ from controls in anxiety-like behavior as measured in the OF test (Fig. 4e–h). Taken together, IO-specific CRF manipulation reveals that IO-CRF plays an important role in challenge-induced motor tuning.

It has been repeatedly reported, mostly using immunostaining and electrophysiology, that CRFR_1_ is expressed in cerebellar PCs^11,15,36–42^. As the main output of the IO is to PCs, we hypothesized that the IO-CRF effect will be driven by PCs. However, we were unable to confirm CRFR_1_ expression in PCs of the CRFR1 reporter line (CRFR_1_^GFP^), using ISH for CRFR_1_ on sagittal mouse brain slices (Supplementary Fig. 4A-E). In contrast, with both methods a signal was detected in molecular layer interneurons (MLIs; red arrows), in the granular cell layer, and in the deep cerebellar nuclei (DCN) as expected (Supplementary Fig. 4B-E).

Next, we used PCP2^cre^ mice, which express *Cre* recombinase solely in PCs, and crossbred them with either floxed CRFR1 mice or floxed CRFR2 mice, (i.e., putative PC-CRFR1_KO_; pPC-CRFR2_KO_). We tested for levels of CRFR1 and CRFR2 mRNA in the cerebelli of this model but no differences were detected in CRFRs mRNA levels in pPC-CRFRs-KO (Supplementary Fig. 4F-G). CRFR_1_ immunohistochemistry on cerebelli from developmental CRFR_1KO_ mice, on the pPC-CRFR_1KO_ mice and WT mice demonstrates that the antibody gives a false signal in PCs. All these mouse lines showed a similar signal in PCs, which was inconsistent with the CRFR_1_-ISH signal and with CRFR_1_^GFP^ reporter line GFP expression (as presented in Supplementary Fig. 4B-C, H).

To conclude, although IO-CRF clearly has a role in motor performance of the mouse, its site of action is not yet clear. We could not validate CRFR1 expression in the PCs of mice, the natural output for IO neurons, and this issue has been noted previously^43–46^. However, IO-CRF may affect MLIs via bulk transmission, or DCN neurons by CF collaterals as both have validated CRFR1 staining, or by an alternative CRFR on PCs (see Discussion).

### IO-CRF is “recruited” in stressful challenges that require motor activity

**Fig. 5:**
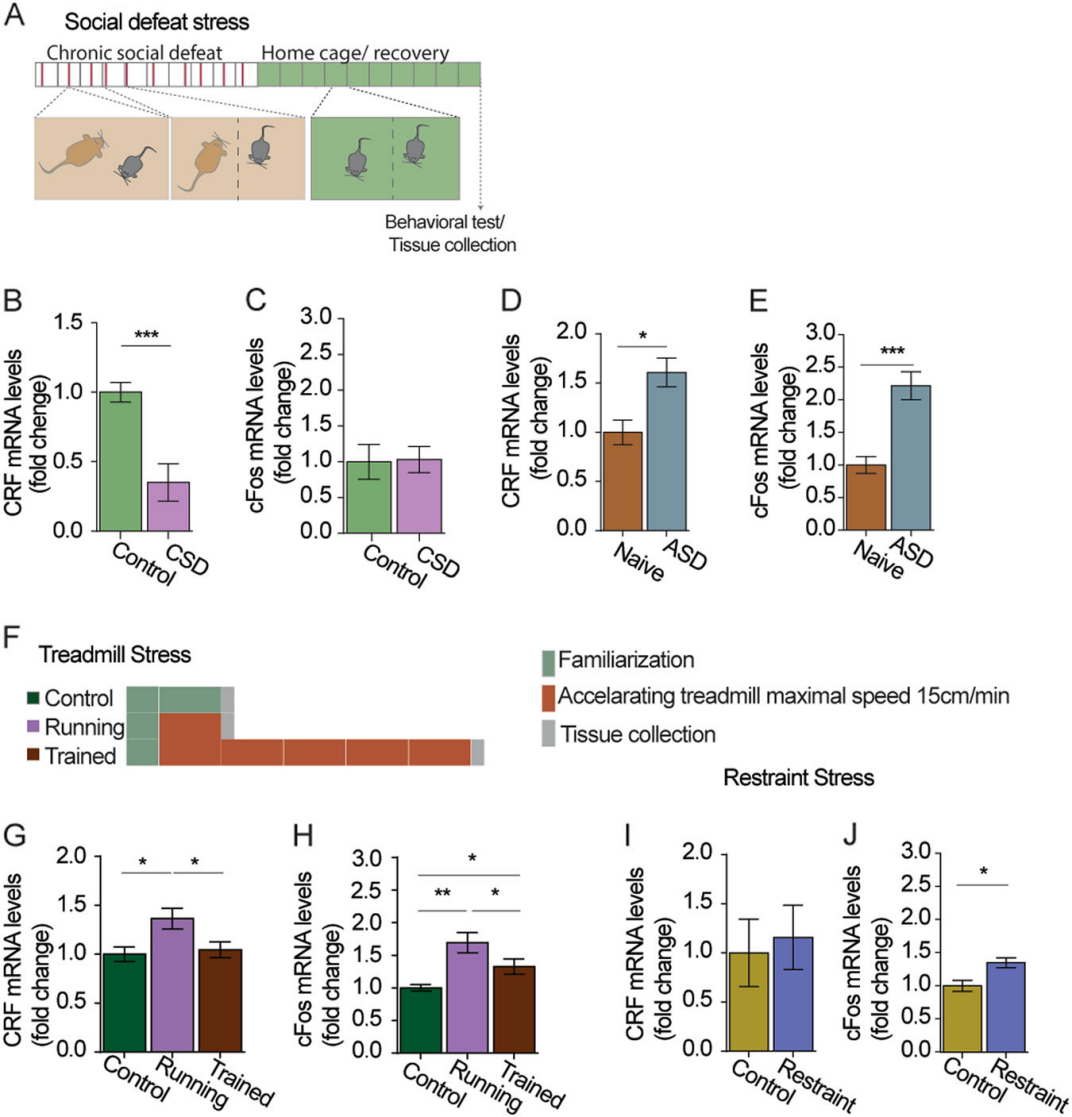
Inferior olive CRF is “recruited” in stressful challenges that require motor activity. **a** Schematic illustration of the chronic social defeat stress (CSDS) paradigm. C57 mice were subjected to 5 min of physical contact with an aggressive ICR mouse (red line), followed by sensory contact for 24 h, for 10 consecutive days following which mice were returned to a cage with their littermates for 10 days. **b** IO-CRF mRNA levels collected 10 days after the end of the CSDS protocol were reduced two-fold compared to controls (*n* = 8, 5). **c** Long-lasting changes in IO cFos levels were not detected in CSDS mice. **d** IO-CRF mRNA levels were increased 90 mins after single acute social stress (ASD) compare to that of control mice (*n* = 8, 7). **e** An increase in IO cFos mRNA levels was also apparent in ASD mice indicating increased IO activity during the defeat (*n* = 8, 7). **f** Schematic illustration of the treadmill protocol used to induce “forced flight response”. Mice were divided into three groups; all groups were familiarized with the apparatus and the shocker 1 day prior to the start of their protocol (green rectangle). One group was trained on the treadmill at a different acceleration pace but similar maximal speed (15 m/min) for 20 min for 4 days (“trained”; brown rectangles). The control group went on the treadmill with the shocker active, but without the treadmill belt moving (i.e., mice did not need to run in order to avoid the shock; “passive avoidance” = “control” group), and the last group was faced with the need to run away from the shocker on test day for the first time (“running” group; single brown rectangle). **g** IO-CRF mRNA levels 90 min after the beginning of the run on an accelerating treadmill were increased only in the “running” group compared to the “trained” and to the “control” group (*n* = 6, 8, 8 for “Control”, “Running”, and “Trained”, respectively). **h** Following treadmill run, cFos mRNA levels differed between all groups. The “running” group showed the highest average cFos mRNA levels, followed by the “trained” group (*n* = 6, 8, 8 for “control”, “running”, and “trained”, respectively). **i** IO-CRF mRNA levels 90 min following the initiation of a single acute restraint stress do not differ from that of control mice (*n* = 5, 5). **j** cFos levels in the IO were mildly increased following acute restraint stress (*n* = 5, 4). Data are presented in mean ± SEM. *p < 0.05, **p < 0.01, ***p < 0.001

Chronic stress, specifically CSDS, can induce long lasting motor impairments^47,48^. To study whether these long-term effects of CSDS correlate with changes in IO-CRF, IO dissections from mice following CSDS were collected and analyzed for CRF mRNA levels using quantitative real-time PCR (qRT-PCR; Supplementary Methods; Fig. 5a). Following CSDS, the mice had an average of 50% lower IO-CRF mRNA levels compared to control mice (*t*_(11)_ = 3.252, *p* = 0.008; *n* = 8, 5; Fig. 5b). Importantly, cFos did not change in CSDS mice, implying there is no long-lasting change in IO activity in chronically stressed mice compared to control mice (Fig. 5c). A different set of mice following a similar CSDS endured less time on the rotarod (Supplementary Fig. 5A) and reached lower maximal velocities (Supplementary Fig. 5B) as reported previously^48^.

The marked long-lasting downregulation of IO-CRF following CSDS raised the hypothesis that during the social defeat, IO-CRF is “recruited” to cope with the challenge. To test this, we exposed C57/BL mice to a single social defeat encounter (acute social defeat, ASD). Measuring mRNA changes shortly after a challenge allows us to detect short-term changes in transcription. These changes are likely a result of restoration of neuropeptide levels following its release. As hypothesized, IO-CRF mRNA was significantly increased shortly following the ASD (Mann–Whitney *U* = 8, *p* = 0.021, *n* = 8, 7; Fig. 5a). To evaluate IO neuronal activity in response to this paradigm, we assessed cFos mRNA levels as well. ASD mice had a significant increase in cFos mRNA (*t*_(13)_ = 4.784, *p* < 0.001; *n* = 8, 7; Fig. 5b).

We further hypothesized that IO-CRF is recruited specifically in stressful challenges that require a well-tuned motor response. We used the accelerating treadmill apparatus to induce a “forced flight response” in the mice, which represents a stressful task that is also inherently motor. Mice were habituated to the treadmill apparatus for 10 min on day 1 while no running was required (Supplementary Methods; Fig. 5c). On the following days, mice were divided into three groups: “control” group was in the apparatus but not running, “trained” group was trained for total of 5 days on the treadmill, and “running” group ran for the first time on test/tissue collection day (Fig. 5c). CRF mRNA showed a marked increase in the “running” group, but not in the “trained” group, compared to controls (one-way ANOVA, *F*_(2,19)_ = 4.206, *p* = 0.031, post hoc Fisher’s LSD running-ctrl *p* = 0.021, trained-running *p* = 0.025; *n* = 6, 8, 8 for “control”, “running” and “trained”, respectively; Fig. 5d). Since CRF mRNA was increased in the “running” group only, it suggests it is independent from IO activity per se, as cFos was increased in both the “running” and “trained” groups compared to “control” (one-way ANOVA, *F*_(2,19)_ = 9.684, *p* < 0.001, post hoc Fisher’s LSD, control-running *p* < 0.001, control-trained *p* = 0.044, running-trained *p* = 0.026; *n* = 6, 8, 8 for “control”, “running”, and “trained”, respectively; Fig. 5e).

Finally, we used restraint stress to determine if a “non-motor” stressful and novel situation results in IO-CRF mRNA regulation. Mice were subjected to immobilization stress shortly followed by tissue collection (see Supplementary Methods). Control mice were left in their home-cages. IO-CRF mRNA did not differ between stressed and control mice, although a small but significant increase in IO cFos was observed (*t*_(7)_ = 3.215, *p* = 0.015, *n* = 5, 4; Fig. 5f, g). Taken together, these results suggest that IO-CRF is important for specific stress induced responses that allow for “motor coping”, but not for all stressful situations.

In this study, we have shown that CRF is expressed throughout the IO and expressed in the majority of IO neurons. Using site-specific KD and partial KO in adult mice, we found reduced levels of CRF resulted in maladaptive motor performance. Lastly, we presented evidence of regulation on IO CRF under specific conditions. This study implicates IO-CRF in the complete adaptive stress response.

## Discussion

In the current study, we examined the role of IO-CRF in mediating stress related motor performance under challenging conditions. In agreement with previous reports, we showed that CRF is highly expressed in the IO and CFs rising from the IO in mice and humans^10,11,14,15,17,49^. The fact that CRF is expressed in the majority IO neurons indicates their importance. However, to date, no study has examined the role of IO neurons in behavior or physiology.

Here we showed that mice with reduced IO-CRF levels presented decreased motor abilities when tested on the rotarod. These experiments indicate that IO-CRF plays a role in the motor capabilities of mice, when faced with a challenge, but does not affect general basal locomotion. Our results are similar to a very recent publication manipulating IO-CRF in rats^50^. Wang and colleagues show that KD rats present lower performance on the rotarod, as well as having some gait disturbance. Our gait analysis results did not yield significant differences (Supplementary Figures 2B and 3A). Nevertheless, on average, pIO-CRFKO showed differences in gait, in a similar direction to those presented in the work by Wang et al. (Supplementary Fig. 3A)^50^. A likely explanation is that the extent of our KD and KO was somewhat smaller, and thus our behavioral effect somewhat milder. Interestingly low to moderate levels of anxiety or CRF in certain brain regions seem to lead to increased locomotion^1^. In our work, a mild increase in locomotion was detected only in the partial IO-CRF_KO_ model (in accordance with “mild stress” situations), which may imply that a strong CRF manipulation may affect locomotion. However further analysis is required in order to conclusively determine a causative link between IO-CRF and locomotion, as it was not detected in the KD mouse model. Nevertheless, it is important to note that generally IO plays a particular role in motor learning and adjustment of movements when facing variable challenges, more so than in baseline locomotion^51,52^. Importantly, IO-CRF manipulation did not affect stress-related behaviors in mice, emphasizing a purely motor role.

Previously published work has established that chronic stress, in particular CSDS, disrupts motor abilities in rodents^47,48,53^. Human studies with PTSD patients revealed that cerebellar connectivity is altered in patients compared to control subjects^20^. Environmental factors linked with depression (including post-stroke depression) were also found to be related to cerebellar connectivity^54^. Cerebellar connectivity was implemented in social anxiety and chronic work stress^22,55^. Moreover, co-morbidity of cerebellar ataxia and depression might involve CRF disfunction^56–58^. However, the direction of the relationship between stress and motor capabilities is not yet clear, and thus further research is needed^21,58^. In our study, we found that defeated mice have a persistent downregulation of IO-CRF mRNA. This implies that maladaptive emotional states are correlated with changes in cerebellar circuitry, which in turn may affect motor related skills, as demonstrated in mice motor performance following CSDS^53^ (Supplementary Fig. 5).

CRF transcript was previously shown to be regulated by different challenges in several brain areas. Moreover, IO-CRF mRNA was specifically shown to be differentially regulated in ataxic mouse lines compared to controls and following harmaline induced tremor and other ataxia models^1,27,59–64^. We further showed that IO CRF mRNA is specifically upregulated shortly after stressful situations that require motor coping. In light of these findings, we hypothesize that IO-CRF is “recruited” specifically when an “enhancement” in coordination/motor capacity is needed in order to efficiently cope with a challenge or stressor.

This hypothesis supports the concept of an upstream mechanism directing different “stress pathways”, as previously suggested^65,66^. Thus far, several brain structures, such as the central amygdala and the periaqueductal gray, have shown differential internal activation that determines whether the behavioral outcome is freezing of fleeting. The differential internal activity in these structures depends, amongst other things, on whether the situation allows for flight or promotes freezing (e.g., if there is no flight route^66,67^). Whether the periaqueductal gray or other structures can regulate CRF release from the IO would be an interesting question to explore in the future.

CFs constitute a direct pathway from the IO to PCs in the cerebellar cortex. The effect of CRF on PCs’ electrophysiological in vitro activity and development have been previously reported^39,40,68–70^, however, the expression of CRFR1 in PCs has also been challenged^43–45^. In spite of the canonical statue of IO-PC pathway, it is important to note that CRFR1 has been reported to be expressed in MLIs and DCN as well^44,71^. These pathways (IO to MLIs, IO to DCNs) are additional plausible pathways through which IO-CRF may affect behavior^50,72,73^. Importantly, a recent publication by Wang et al., indeed shows that CRF injection to the DCN ameliorates IO-CRFKD induced motor phenotype^50^. Besides, MLIs, although traditionally affected by granular cells’ parallel fibers, do not have any other likely input of CRF except for the IO. This further supports the likelihood of IO-CRF affecting via bulk transmission^74^. Future research should examine the effect of CRFR1 manipulations in the various cell types likely to be affected by CRF. Moreover, genetic studies including single cell sequencing are needed to further assess the expression of CRFRs in PCs, and their extent. To conclude, IO-CRF participates in the IO-cerebellar pathway and affects the organisms’ motor capabilities under specific conditions. We are yet to determine what the upstream and downstream mechanisms involved are, as well as the cellular mechanisms involved.

It is important to note the human aspect, as CRF is highly expressed in the human IO, and PTSD and stress related pathology patients show altered cerebellar connectivity^20–22,55^. Therefore, it is essential to consider and further explore if chronic stress has an impact on the ability to physically cope with challenging situations. This aspect has been mostly disregarded in the field of stress-neurobiology, despite basic central motor mechanisms being as important as emotional mechanisms in an organism’s survival.

## Electronic supplementary material

Movie 1. IO-CRF neurons projecting to the cerebellum

Supplemental Information

Supplementary Figure 1

Supplementary Figure 2

Supplementary Figure 3

Supplementary Figure 4

Supplementary Figure 5
