# Supplementary material for: Inferior olive CRF plays a role in motor performance under challenging conditions
Source: Transl Psychiatry. 2018 May 25;8:107. doi: 10.1038/s41398-018-0145-3 (PMC5970254; doi:10.1038/s41398-018-0145-3)
Supplement: Supplementary file 2 — Supplemental Information [file 41398_2018_145_MOESM2_ESM.docx]

## Supplemental Information

## Material and Methods

**Animals and Experimental Groups.**

All animals were housed in a temperature-controlled room (22 ± 1 °C) on a reverse 12 h light/dark cycle. Experiments were conducted on 8-15-week-old male mice during the dark phase. Food and water were given *ad libitum*. Experimental protocols were approved by the Institutional Animal Care and Use Committee of the Weizmann Institute of Science.

For CRF reporter line mice expressing *Cre*-recombinase under the CRF promoter (CRH-ires-*Cre*; Jackson laboratories, Bar Harbor, ME; <http://jaxmice.jax.org/strain/012704.html>) were crossed with mice conditionally expressing tdTomato (1) Jackson laboratories, Bar Harbor, ME). The CRFR1 reporter line we used expressed GFP under the CRFR1 promoter (CRFR1^GFP^; (2) <http://jaxmice.jax.org/strain/007909.html>). For the IO-CRF knock out (KO) mouse line, floxed CRF mice were generated by classical gene targeting procedures in mouse embryonic stem cells (TBV2; 129S2/SvPas). LoxP sites were placed in intron 1 and in the 3´UTR of exon 2. The floxed allele is functionally indistinguishable from the wild-type CRF allele (Detailed description is available upon request). Mouse genotypes were determined using polymerase chain reaction (PCR). The PC-specific CRFR knock-out (pPC-CRFR_KO_) was generated by crossing mice carrying a conditional allele of CRFR_1_ (3) or CRFR_2_ generated in our lab with mice expressing *Cre*-recombinase under PCP2 promoter PCP2-*Cre* (pcp2*-Cre*, Jackson Laboratories, West Grove, PA, USA; [https://www.jax.org/strain/004146](https://www.jax.org/strain/004146%2054) ) (4).

## **Behavioral Studies**

The experimenter was blind to group allocation during behavioral experiments by using mock numbering for the animals. Behavioral data was collected by automated means, and whenever possible, complete automated analysis was used.

*Rota-rod*: Mice were placed on a standard rota-rod apparatus (San Diego Instruments, San Diego, CA) with linearly increasing speed from 0 to 40 rpm over a 4-min period or over a 2-min period (which results in a 10 or 20 rpm inclination per min). Mice had a 5-min habituation period on the apparatus immediately followed by 4 consecutive trials, on the rotating cylinder. Mice were given a 2-min inter-trial break. The sum of the latencies to fall off the rotating cylinder and maximal velocity reached in the session were calculated.

*Home-cage locomotion*: Locomotion was assessed using the InfraMot system (TSE Systems, Bad Homburg, Germany). Mice were housed individually for 72 h. Measurements of general locomotion consisted of 2 x light and 2 x dark cycles collected at 30 min intervals (excluding day 1).

*Open-field (OF) test*: This test was performed as previously described (5,6). The OF test was performed in a 50 x 50 x 22 cm white box, lit to 120 lux. The mice were placed in the box for 10 min. Locomotion in the box was quantified using a video tracking system (VideoMot2; TSE Systems, Bad Homburg, Germany).

*Treadmill*: Treadmill apparatus (Panlab, Harvard apparatus, Cornella, Spain; LE8710M) consisted of a rolling belt with adjustable speed and acceleration. The apparatus provides an electrical shock from a grid situated at the end of the rolling belt. Mice were first familiarized for 10 mins with the shocker operating at 0.2 mA, while the treadmill belt was not moving. During habituation, mice were allowed to freely explore the apparatus and familiarize with the shocker. The test day, mice were subjected to the treadmill accelerating according to a crescendo protocol: during the first 10 mins, the speed was increased from 5 m/min to 10 m/min while in the following 5 mins, speed was increased from 10 m/min to 15 m/min. Trained animals were subjected to similar maximal speed (15 m/min) for 4 additional days, but with a different acceleration protocols (i.e., they could not learn the exact pace of running needed to avoid the shocker). All runs lasted 20 min, all tissues were collected 90 mins after the start of the run/task.

*Catwalk:* For automated gait analysis, we used CatWalk apparatus (Noldus Information Technology bv, Wageningen, Nethelands). The CatWalk consists of a glass plate suspended 1.5 m above ground, a black corridor structuring the ‘runway’. Underneath the ‘runway’ is a camera collecting data on all footprints generated by the mouse on the catwalk. Each mouse was placed on one end of the corridor and left to run back and forth along the runway until completing 5 compliant runs, and then returned to its home cage. Data about Stride length (distance between successive prints on the same side), base of support (DOB; distance between hind and front paws) and Regularity index (percentage of footprints that are part of the regular walking pattern) measures were collected to have an overall assessment of postural imbalance and coordination.

*Chronic social defeat stress:* Briefly, the mice were placed in the home-cage of an aggressive ICR mouse and allowed to physically interact for five minutes, during which the ICR mouse attacked the intruder mouse. A perforated clear Plexiglas divider was then placed between the mice and they remained in the same cage for 24 hours to allow sensory contact, this procedure was repeated for 10 days. Tissue collection was done following an additional 10 days of recovery. Control mice were kept in similar conditions but did not encounter a bully mouse (as described in (7)).

*Acute social defeat stress*: Mice were subjected to a single 10-min physical interaction and an additional 20 min with a mesh separation (total of 30 min stress induction), following which mice were returned to their home-cage. Tissue was collected 90 min after the beginning of the defeat.

*Restraint stress:* Restraint stress was induced by putting the mice into a ventilated 50 ml plastic conical tube for 30 min. Naïve mice were used to establish basal transcript levels. Tissue was collected 90 min from the beginning of the stress/task.

**Immunohistochemistry and *In-situ* hybridization**

For immunohistochemistry, *in-situ* hybridization (ISH), and clarity, animals were anesthetized and perfused with phosphate buffered saline (PBS) followed by 4% PFA.

**Immunohistochemistry**: Floating sections (30-50μm-thick) were permeabilized with 0.2% triton for 30 min, followed by blocking with 20% normal horse serum (NHS) and 0.5% triton in PBS for 2 hours then incubated with the primary antibody (1:200-1:1000, according to the manufacturers’ recommendations) diluted in PBS with 2% NHS and 0.1% triton, overnight. Slices were then washed in PBS, followed by incubation with 1:200 secondary antibody for 1-2 hours. Slices were washed and then briefly incubated with Hoechst 1:10000, washed and mounted on slides and covered with a cover slip. When required, the M.O.M kit blocking reagent was used in place of NHS (Vector laboratories, Burlingame, CA, USA).

**Digoxigenin *In-situ* hybridization**

The procedure used for ISH was as described previously (8). Briefly, ISH was carried out with the free-floating section method, as follows. Antisense RNA probes were generated using rat CRFR1 cDNAs (kindly provided by Dr. W.W. Vale, San Diego, CA, USA) and labeled with DIG-11-UTP using a labeling kit (Roche Molecular Biochemicals, Basel, Switzerland). All ISH steps were carried out at room temperature unless otherwise stated. The alkaline phosphatase method was used with nitro-blue tetrazolium chloride/5-bromo-4-chloro-3-indolyl phosphate, toluidine salt (NBT/BCIP) as the substrate for the detection of the DIG label. Sections were mounted on glass slides and cover-slipped with Entellan. Images of mounted slices were obtained with a light microscope, and the relative DIG signal coverage of IO region was calculated in each section (2-3 per mouse) blindly using Fiji software (https://fiji.sc/).

**Radioactive *In situ* hybridization.**

The radioactive *in situ* hybridization procedure used has been described elsewhere (9). Briefly, animals were decapitated, brains were removed and frozen on dry ice. 20 μm coronal sections were thaw mounted onto glass slides, and stored at -80°C until use. Antisense cRNA probes were transcribed from linearized cDNA templates corresponding to CRF (1200 bp). [35S]UTP (PerkinElmer, Emeryville, CA) was incorporated into cRNA probes during transcription. Brain sections were fixed in 4% paraformaldehyde and treated with 0.25% acetic anhydride in 0.1M triethanolamine, pH 8.0, followed by a 2% SSC rinse, dehydrated through a graded series of alcohols, delipidated in chloroform, rehydrated through another series of alcohols, and then air dried. The slides were exposed overnight to cRNA probes in humidified chambers at 55 °C. Following incubation, slides were washed in SSC of increasing stringency, in RNase, and then in 0.1% SSC at 63°C, dehydrated through a graded series of alcohols, and dried. Slides were dipped in NTB-2 emulsion (Eastman Kodak, Rochester, NY), exposed from day 7 to 14 at 4 °C, and developed. After development, the slides were counterstained with cresyl violet. Autoradiograms for hybridization were visualized under dark-field illumination using a E600 light microscope (Nikon, Tokyo, Japan).

**Antibodies**

Primary antibodies: The following primary antibodies were used: Anti-GFP antibody (1:200; both biotinylated and non-biotinylated were used according to need) raised in goat or rabbit (AB_303395, AB_304897; Abcam, Cambridge, UK). Rabbit anti-CRF (1:1000; kindly provided by Dr. Wylie Vale, The Salk Institute). Monoclonal Anti-Calbindin-D-28K antibody produced in mouse (1:500; AB_476894; Sigma-Aldrich, St. Louis, MO), anti-*Cre* (1:1000; clone 2D8; AB_2085748) and anti-NeuN raised in mouse (1:50; AB_2298772; Millipore, Billerica, MA) and anti-mouse biotinylated when needed (1:200; AB_2336564; Vector laboratories, Burlingame, CA). For CRFR_1/2_ staining we used CRFR_1/2_ antibody (C-20) raised in goat (1:1000; AB_673600; Santa Cruz, Dallas, TX, USA).

Secondary Abs: As a secondary antibody, we used anti goat, rabbit or mouse Alexa 488 (1:200; Invitrogen Life Technologies, Carlsbad, CA). When needed, Alexa streptavidin conjugated 647 or 488 were used (1:200; Invitrogen Life Technologies, Carlsbad, CA).

Identification of NeuN and CRF colocalization was done by eye with the assistance of Fiji software (<https://fiji.sc/)>.

**CLARITY and Whole-Brain Imaging**

Brain clearing protocol was based on a modified CLARITY protocol (10) with few changes. Briefly, CRF-Cre x Ai9 mice were transcardially perfused with PBSx1 followed by ice-cold 4% PFA. Brains were post-fixed in 4% PFA overnight at 4°C and then transferred to 1% hydrogel solution (1% acrylamide, 0.125% Bis-Acrylamide, 4% PFA, 0.025% VA-044 initiator, in PBSx1) for 72 hours. Brains were then degassed and polymerized for 5 hours at 37°C. Next, whole brains were cut into 2 hemispheres and washed with 200mM NaOH-Boric buffer (pH=8.5) containing 8% SDS for 12 hours. Brain hemispheres were transferred to a clearing device built from a temperature-control circulator, and containing clearing solution (100mM Tris-Boric Buffer, 8% SDS at 37-40°C). Twelve days later the samples were washed in PBST (0.2% Triton- X100) for at least 48-72 hours at 37°C. Brain hemispheres were incubated for 2-4 days in a refractive index matching solution (RIMS;(11)) containing phosphate buffer and Histodenz (Sigma–Aldrich, Wicklow, Ireland). Brain hemispheres were then imaged using light-sheet microscope (Ultramicroscope II, LaVision BioTec GmbH, Bielefeld, Germany). Briefly, brain hemispheres were mounted on a holder and the imaging chamber was filled with 100ml of RIMS. Imaging was done using a 2x/0.5NA objective at 1x magnification, while both light sheets illuminating the sample, and the Z-step was set to 5 μm. 16-bit TIFF files were stitched and visualize using arivis Vision4D software (arivis, Unterschleißheim, Germany).

**Viral constructs and stereotaxic injections**

Viruses were injected using a computer-guided stereotaxic instrument and a motorized nano-injector (Angle Two^TM^ Stereotaxic Instrument, myNeurolab, Leica Biosystems, Buffalo Grove, IL). Mice were placed on a stereotaxic apparatus under general isoflurane anesthesia (AbbieVie ltd, Maidenhead, England). The virus preparation was delivered using a Hamilton syringe connected to the motorized nano-injector system. The solution was injected at a rate of 0.125μl per min. For IO-CRF_KD_ experiments, lentivirus expressing shCRF and control viruses, (described in (6,7)) were injected bilaterally to C57/BL mice (1μ vol, AP-7 mm, ML +0.5 mm, DV-6 mm). Following a 2-week recovery period, mice were subjected to behavioral tests. For the partial IO-CRF_KO_, AAV8-CMV-*Cre*ERT2 (VectorBiolabs, Malvern, PA, USA) or control virus, AAV8-CMV-GFP were injected to the adult floxed CRF mice, or WT littermates, bilaterally into the IO (0.4 μl vol, AP-7 mm, ML +0.2 mm, DV-6 mm). *Cre*-ERT2 enables a time restricted induction of *Cre* entrance into the cell’s nucleus to induce recombination due to the mutated estrogen receptor (ERT). The *Cre*-ERT2 can only enter the nucleus when it is bound to tamoxifen. This reduces any adverse side effects of *Cre* to a minimum. Injected mice were given a 2-week recovery period followed by only 2 repeats of gavage tamoxifen delivery (5mg/mouse dissolved in 100μl solution comprised of 10% ethanol and 90% peanut oil).

For CRF immunostaining (Fig. 1E-G), mice were injected with colchicine (2 μl of 1 μg/μl dilution) into the lateral ventricles (AP −0.20 mm; ML +0.95 mm; DV −2.2 mm). Mice were killed by perfusion once locomotor symptoms were observed (3–4 days following col­chicine injection).

At the end of the behavioral experiments, tissue was collected from all mice in order to evaluate the location accuracy of viral injection. Mice that did not show any viral signal in the IO were excluded from the behavioral analysis.

## **mRNA extraction and quantification with qRT-qPCR**

Immediately after decapitation, the brain was removed and the IO or cerebelli dissected and stored at -80 °C until further analysis. For dissected IO, RNA extraction was carried out using miRNeasy mini kit (QIAGEN, Hilden, Germany) according to the manufacturer's instructions. Whole cerebelli RNA extraction was performed using Tri-reagent® according to the manufacturer’s protocol (Sigma-Aldrich®, St. Louis, MO). Reverse transcription was done using high capacity RT-kit (Applied Biosystems, Carlsbad, CA). cDNA samples were then analyzed using SYBR^®^Green PCR kit (QIAGEN, Hilden, Germany) according to the manufacturer's guidelines in StepOne™ thermocycler (Applied Biosystems, Waltham, MA) for CRF (F: 5’ GCAGTTAGCTCAGCAAGCTCAC 3’, R: 5’ CAAATGATATCGGAGCTGCG 3’), cFos, (F: 5’ ATCCGAAGGGAACGGAATAAGA 3’, R: 5’GTTGATCTGTCTCCGCTTGGA 3’) and HPRT, which was used as an internal control (F: 5’ GCAGTACAGCCCCAAAATGG 3’, R: 5’GGTCCTTTTCACCAGCAAGCT 3’).

Supplementary figures

**Movie 1. IO-CRF neurons projecting to the cerebellum.**

The movie shows maximum intensity projection of a brain hemisphere taken from a CRF-Cre x tdTomato mouse model and processed using a modified CLARITY protocol and imaged by light-sheet microscopy. CRF expressing neurons projects heavily from the IO (located just below the bottom right part of the image) to the cerebellum. In addition, CRF expressing neurons can be visualized in many other brain regions known to express CRF (e.g., cortex, hypothalamus, amygdala).

**Supplementary figure 1:**

**CRF is expressed throughout the IO**

A) Schematic coronal representation of the IO and its sub-nuclei. IOpr= IO principal nucleus; IOD= IO dorsal nucleus; IODM= IO dorsomedial cell group; IOM=IO medial nucleus; IODMC=IO dorsomedial cell column; IOK= cap of Kooy medial nucleus; IOBe = IO beta subnucleus; IOV= IO ventral nucleus; IOB =IO subnucleus B medial nucleus; IOC=IO subnucleus C medial nucleus; IOA=IO subnucleus A medial nucleus.

B) The IO of a CRF-Ai9 reporter line indicates CRF is expressed throughout the IO.

C) CRF *in-situ* hybridization shows CRF expression patterns throughout the IO similarly to the reporter line.

*images presented in figure 1.

D) Representative image of CRF-Ai9 mouse IO stained with an IO marker (Calbindin; green). IOD= IO dorsal nucleus; IOK= cap of Kooy medial nucleus; IOBe= IO beta subnucleus; IOC= IO principal nucleus; IOM= IO medial nucleus; IOpr= IO principal nucleus.

F) Representative images of CRF-Ai9 mouse IO cell bodies expressing tdTomato, immunostained for calbindin (green), and a merged image. Yellow scale = 100 μm.

G) Percentage of IO tdTomato (CRF) positive cells stained for calbindin indicate a large percentage (more than 70%) of IO cells express CRF (based on the staining of 4 sections from 2 mice, a total of 8 sections, each dot in the graph represents a quantified IO image).

**Supplementary figure 2:**

**Mice with an IO specific CRF KD show intact gait**

A) Schematic illustration of gait analysis using the CatWalk. B) IO-CRF_KD_ mice did not show any postural imbalance compared to control mice on the CatWalk apparatus (n=8,9). No change in baseline coordination was detected in IO-CRF_KD_ compared to controls (n=8,9).

**Supplementary figure 3:**

**A mild effect on gait was detected in mice with partial IO-CRF KO**

A) Posture of pIO-CRF_KO_ and control mice was tested using the CatWalk. On average pIO-CRF_KO_ presented a higher distance between the front and the hind paws compared to controls, however this difference was not statistically significant after correction for multiple comparisons (n=10,8). B) Regularity index did not differ between pIO-CRF_KO_ and control mice (n=10,8).

**Supplementary figure 4:**

**CRF receptor type 1 was not detected in PCs.**

A) Schematic illustration of a sagittal slice of mouse brain used to fully visualize the Purkinje cell’s (PCs) dendritic tree. B) CRFR_1_ seem to be expressed in low to moderate levels in the molecular layer (MLI), as seen using CRFR_1_-GFP reporter mouse line (green) but it is not detected in the mouse PCs (stained for calbindin; cyan). C) *In-situ* hybridization shows a similar pattern of CRFR_1_ expression in MLIs, but not PCs (cyan). Red arrows indicate CRFR_1_-GFP/CRFR_1_ *in-situ* hybridization signal; gray arrows indicate PC location; yellow scale bar = 100 μm. D) CRFR_1_ is detected in the mouse deep cerebellar nuclei (DCN) in CRFR_1_-GFP reporter mouse line (green) and by E) *in-situ* hybridization against CRFR_1_. Red arrows indicate CRFR1-GFP/CRFR_1_ *in-situ* hybridization signal. Yellow scale bar = 100 μm. F) Schematic illustration of the cerebellum. Cells expressing CRFR1 are depicted in green shades, cells/fibers expressing CRF in red-pink shades. PC= Purkinje cells, GrC = granular cells, MLI= molecular layer interneurons, IO = inferior olive, DCN = deep cerebellar nuclei. G) PCP2-*Cre* mouse line was crossed with floxed CRFR_1_ or CRFR_2_ mice lines, to obtain Purkinje cells specific CRFR knockout (putative PC-CRFR_KO_; pPC-CRFR_KO_). H) CRFRs qRT-PCR levels from the cerebelli of pPC-CRFR_KO_ levels of CRFRs in pPC-CRFR_1KO_ mice or PC-pCRFR_2KO_ did not differ from controls. I) The commonly used CRFR_1_ antibody was used to detect CRFR_1_ expression in PCs of WT mice, PC-pCRFR_1KO_ and developmental CRFR_1KO_. All 3 mouse lines had cerebellar CRFR_1_ positive staining, indicating the antibody against CRFR_1_ is not reliable. Gray arrows indicate PCs location.

**Supplementary figure 5:**

**Chronic social defeat stress results in long term challenge-induced motor impairment**

1. Following CSDS, mice lasted less time on the rotarod compared to control mice (repeated measures ANOVA, main effect for group, F_(1,23)_= 6.022, p= 0.022, n=12,13). B) CSDS mice reached lower maximal velocities compared to control mice (repeated measures ANOVA, main effect for group, F_(1,23)_= 5.208, p= 0.032, n=12,13; Fig. 2D).

Supplementary bibliography

1. Madisen L, Zwingman TA, Sunkin SM, Oh SW, Zariwala HA, Gu H, et al. A robust and high-throughput Cre reporting and characterization system for the whole mouse brain. Nat Neurosci. 2010 Jan;13(1):133–40.

2. Justice NJ, Yuan ZF, Sawchenko PE, Vale W. Type 1 corticotropin-releasing factor receptor expression reported in BAC transgenic mice: Implications for reconciling ligand-receptor mismatch in the central corticotropin-releasing factor system. J Comp Neurol. 2008 Dec 1;511(4):479–96.

3. Kühne C, Puk O, Graw J, Hrabě de Angelis M, Schütz G, Wurst W, et al. Visualizing corticotropin-releasing hormone receptor type 1 expression and neuronal connectivities in the mouse using a novel multifunctional allele. J Comp Neurol. 2012 Oct 1;520(14):3150–80.

4. Barski JJ, Dethleffsen K, Meyer M. Cre recombinase expression in cerebellar Purkinje cells. Genes N Y N 2000. 2000 Dec;28(3–4):93–8.

5. Volk N, Pape JC, Engel M, Zannas AS, Cattane N, Cattaneo A, et al. Amygdalar MicroRNA-15a Is Essential for Coping with Chronic Stress. Cell Rep. 2016 Nov 8;17(7):1882–91.

6. Regev L, Tsoory M, Gil S, Chen A. Site-specific genetic manipulation of amygdala corticotropin-releasing factor reveals its imperative role in mediating behavioral response to challenge. Biol Psychiatry. 2012 Feb 15;71(4):317–26.

7. Elliott E, Ezra-Nevo G, Regev L, Neufeld-Cohen A, Chen A. Resilience to social stress coincides with functional DNA methylation of the Crf gene in adult mice. Nat Neurosci. 2010 Nov;13(11):1351–3.

8. Korosi A, Veening JG, Kozicz T, Henckens M, Dederen J, Groenink L, et al. Distribution and expression of CRF receptor 1 and 2 mRNAs in the CRF over-expressing mouse brain. Brain Res. 2006 Feb 9;1072(1):46–54.

9. Chen A, Zorrilla E, Smith S, Rousso D, Levy C, Vaughan J, et al. Urocortin 2-deficient mice exhibit gender-specific alterations in circadian hypothalamus-pituitary-adrenal axis and depressive-like behavior. J Neurosci Off J Soc Neurosci. 2006 May 17;26(20):5500–10.

10. Ye L, Allen WE, Thompson KR, Tian Q, Hsueh B, Ramakrishnan C, et al. Wiring and molecular features of prefrontal ensembles representing distinct experiences. Cell. 2016;165(7):1776–1788.

11. Treweek JB, Chan KY, Flytzanis NC, Yang B, Deverman BE, Greenbaum A, et al. Whole-body tissue stabilization and selective extractions via tissue-hydrogel hybrids for high-resolution intact circuit mapping and phenotyping. Nat Protoc. 2015 Nov;10(11):1860–96.
