# Supplementary figures and images for: Inferior olive CRF plays a role in motor performance under challenging conditions
Source: Transl Psychiatry. 2018 May 25;8:107. doi: 10.1038/s41398-018-0145-3 (PMC5970254; doi:10.1038/s41398-018-0145-3)

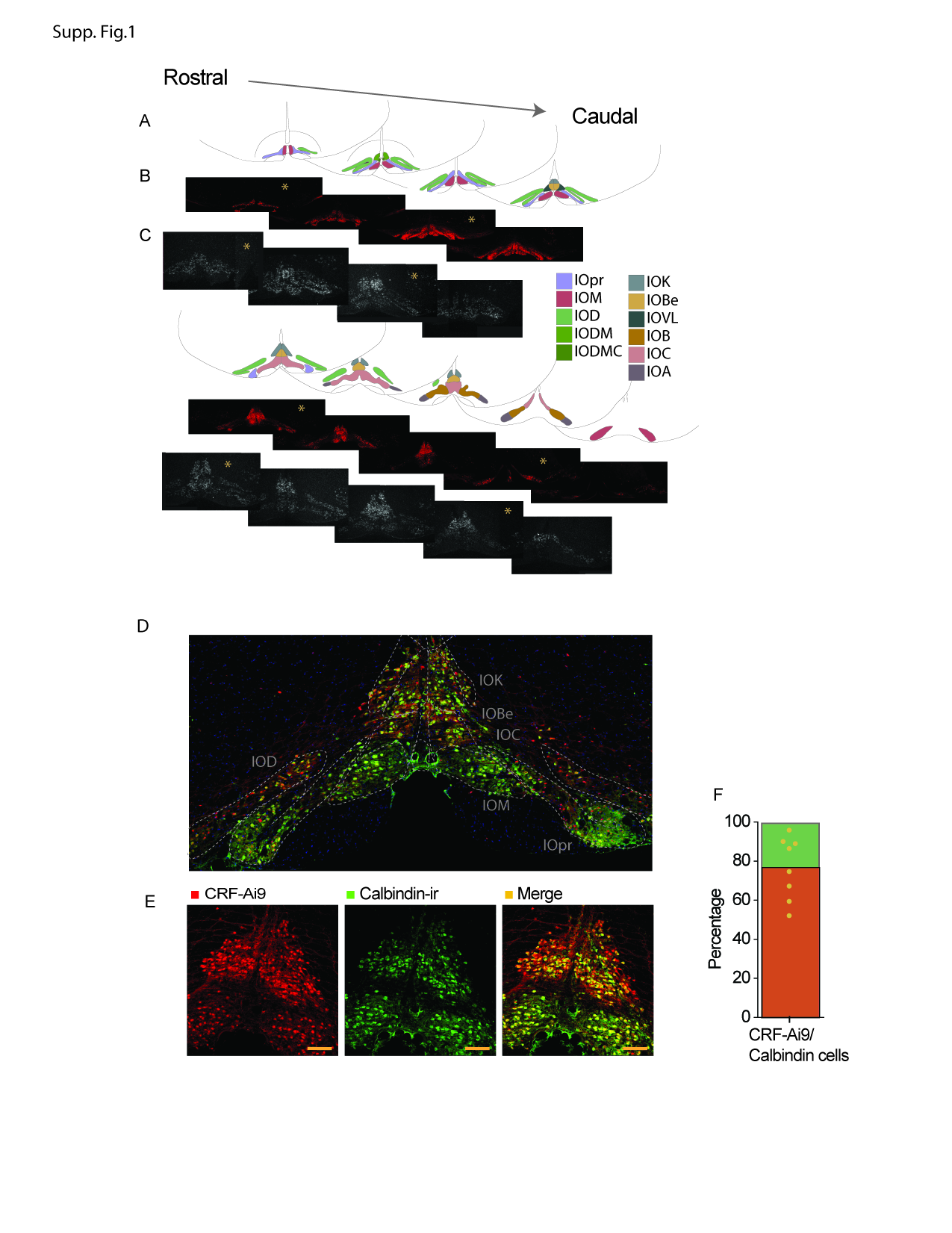

Supplement: Supplementary file 3 — Supplementary Figure 1 [file 41398_2018_145_MOESM3_ESM.tif]

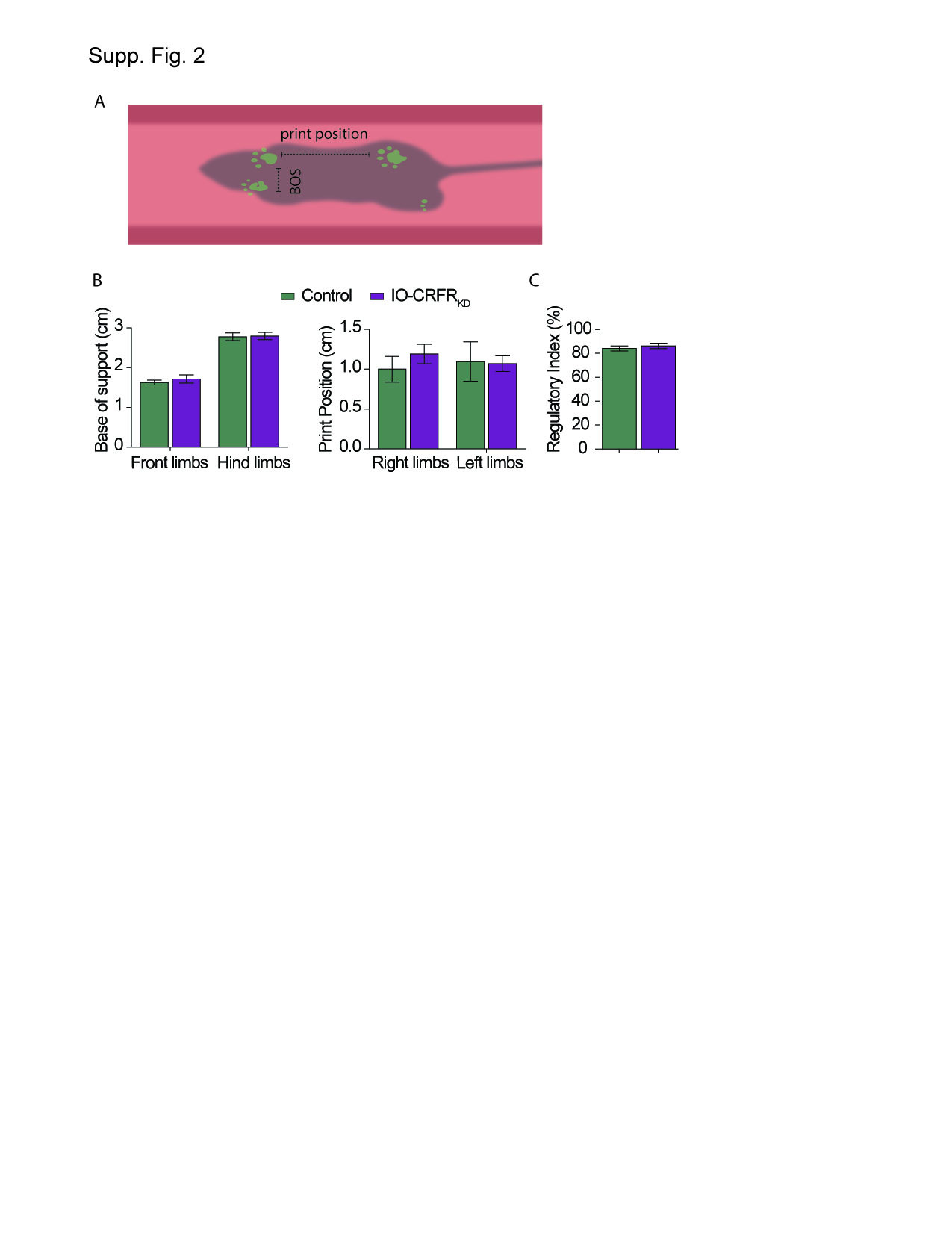

Supplement: Supplementary file 4 — Supplementary Figure 2 [file 41398_2018_145_MOESM4_ESM.tif]

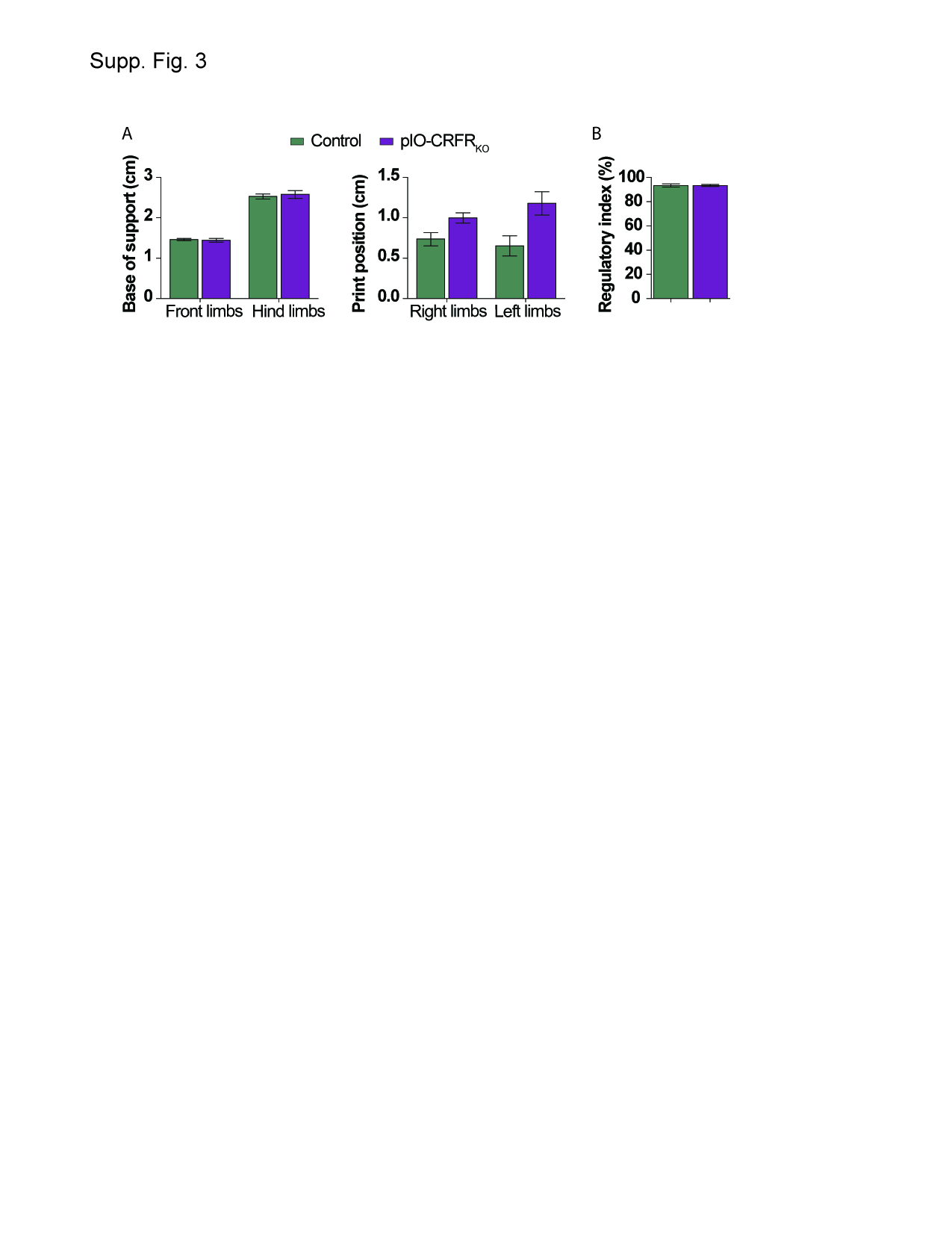

Supplement: Supplementary file 5 — Supplementary Figure 3 [file 41398_2018_145_MOESM5_ESM.tif]

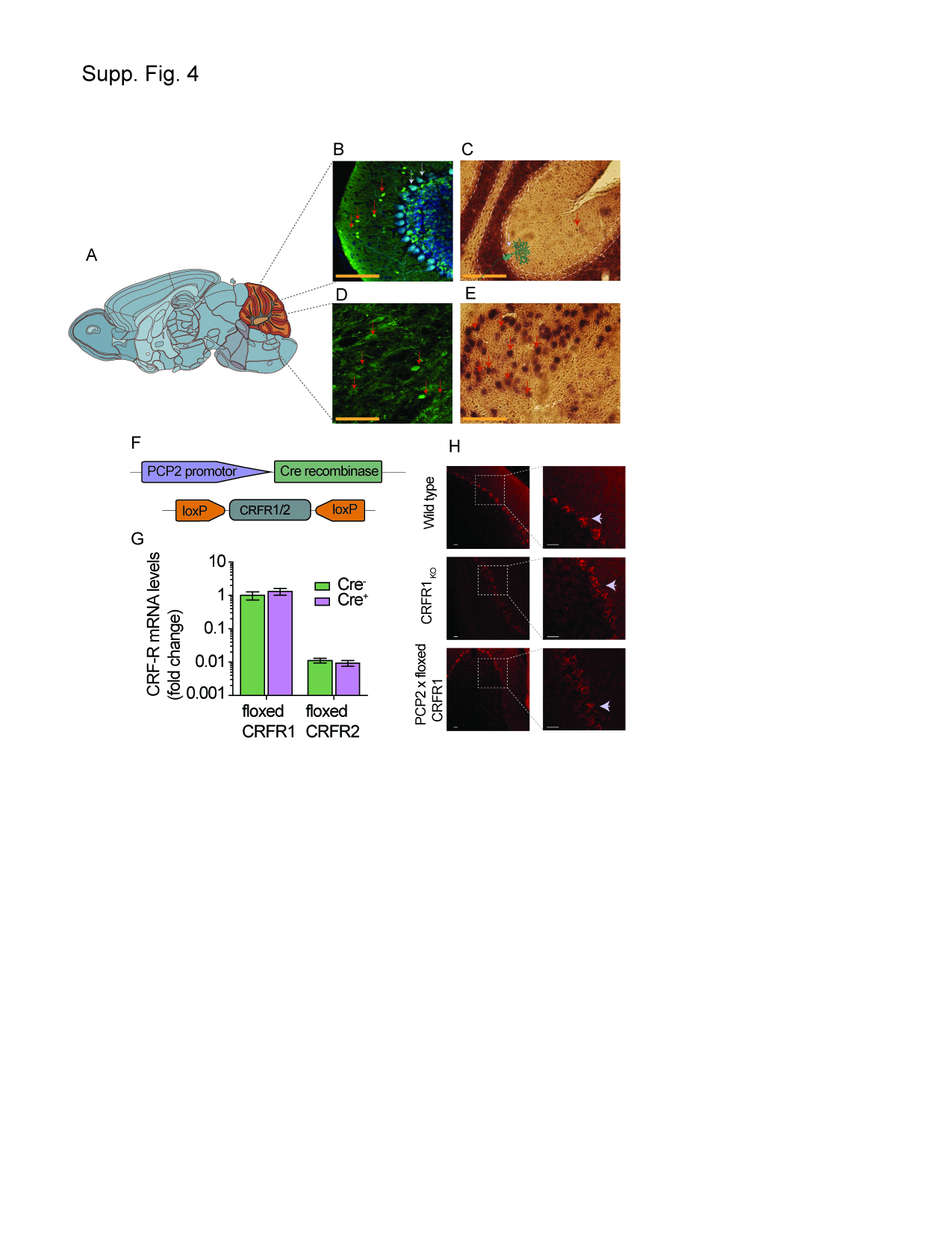

Supplement: Supplementary file 6 — Supplementary Figure 4 [file 41398_2018_145_MOESM6_ESM.tif]

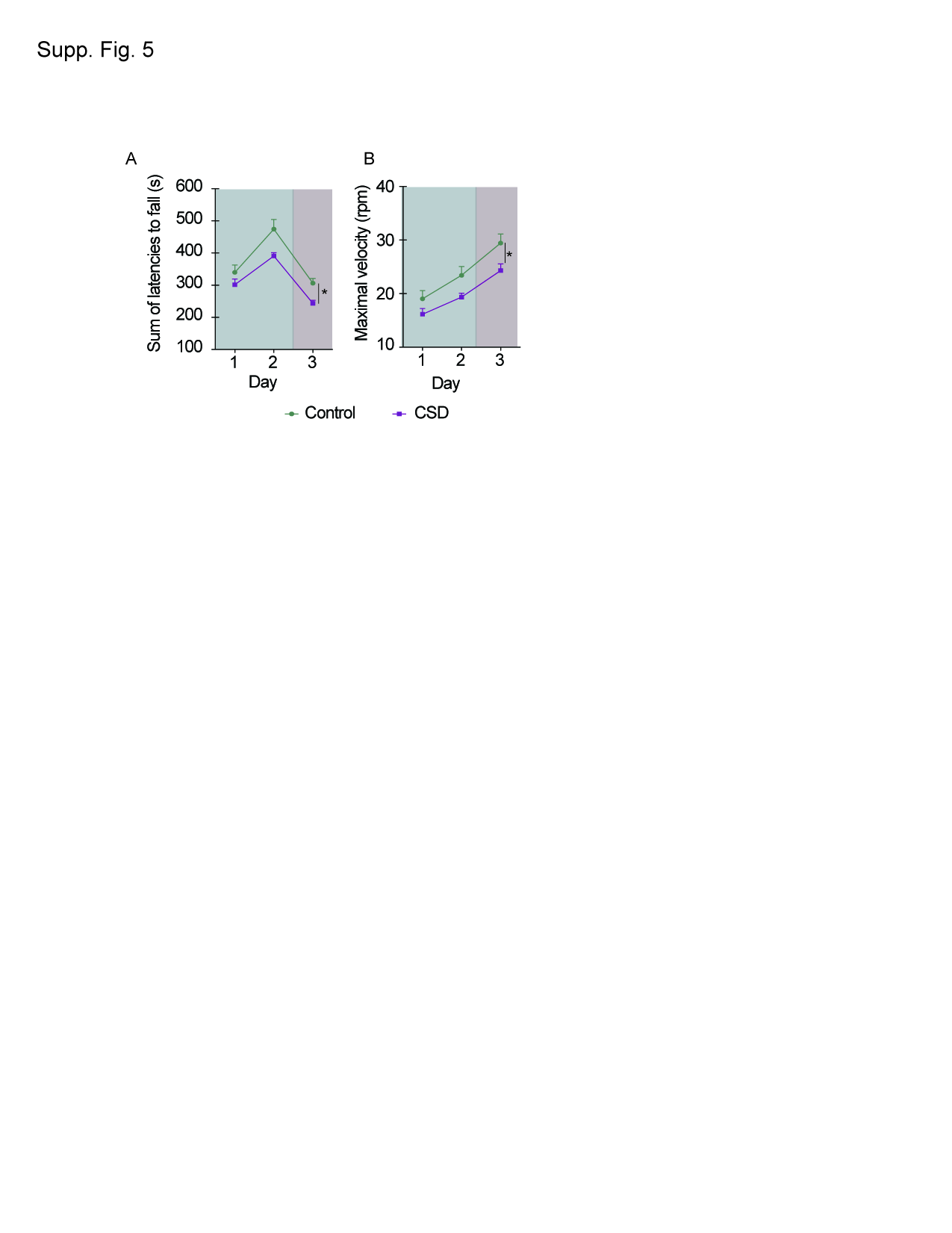

Supplement: Supplementary file 7 — Supplementary Figure 5 [file 41398_2018_145_MOESM7_ESM.tif]
